# The Effect of Cancer and Cancer Treatment on Pubic Symphysis Age Estimation Using Computed Tomography Scans

**DOI:** 10.3390/diagnostics14141500

**Published:** 2024-07-12

**Authors:** Maya N. Alibrio, Sean D. Tallman

**Affiliations:** 1 Department of Anatomy and Neurobiology, Boston University Chobanian and Avedisian School of Medicine, Boston, MA 02118, USA; malibrio@bu.edu; 2 Department of Anthropology, Boston University, Boston, MA 02215, USA; 3 Department of Human Biology, Division of Clinical Anatomy and Biological Anthropology, University of Cape Town, Cape Town 8001, South Africa

**Keywords:** forensic anthropology, Suchey–Brooks method, pubic symphysis, biological profile, computed tomography scans, cancer

## Abstract

It is currently unknown whether cancer and cancer treatment affect age-related skeletal changes used in the biological profile for skeletonized remains. This research examines the effects of cancer on skeletal age estimation using computed tomography (CT) scans of the pubic symphyses for 307 individuals from the New Mexico Descendent Image Database. The Suchey–Brooks method was applied to 125 individuals without documented cancer and 182 individuals with documented cancer. Individuals were correctly aged if their chronological age fell within the original study’s 95% prediction range. Though not statistically significant, the results show that females with cancer were aged correctly 74.7% of the time, and females without cancer were aged correctly 85.1% of the time; males with cancer were aged correctly 46.0% of the time, and males without cancer were aged correctly 55.7% of the time. Additionally, a total of 30 individuals were reanalyzed to examine intraobserver error, and a Cohen’s kappa value of *k* = 0.600 indicated a moderate level of agreement. While no statistical differences were found between cancer and control groups, CT scans may lack the resolution needed to visualize the nuanced effects of bone mineral density loss, if present, and the overall quality of bone, despite their proven utility in dry-bone skeletal analyses.

## 1. Introduction

### 1.1. Cancer and Bone Mineral Density Loss

The World Health Organization (WHO) reports that the four most common cancers worldwide are breast, lung, colorectal, and prostate [1]. Abundant research indicates that cancer and cancer treatment can affect bone mineral density (BMD), which can lead to osteoporosis in both assigned females at birth (AFAB) and assigned males at birth (AMAB) [2,3,4,5]. Osteoporosis is defined as a systemic skeletal disease characterized by low bone mass with a deterioration of bone tissue and a subsequent increase in fragility and fracturing [6]. One of the most prevalent ways in which BMD can decrease due to cancer is through a process known as hypogonadism [4]. This is a condition where the sex organs cease the production of hormones (i.e., estrogen, androgen, and testosterone), whether due to radiation treatments or cancerous tumors. Sex hormones play a fundamental role in regulating BMD. For example, estrogen acts through two different mechanisms—direct and indirect—to control bone resorption while an individual is young [3]. At the onset of menopause, the reduction of estrogen production in the ovaries results in an increase in osteoclastic cell formation with little new growth of osteoblastic cells, which is one of the major causes of osteoporosis and low BMD in AFABs [3]. In AMABs, androgens play a critical role in the preservation of BMD, comparable to estrogen in AFABs. As AMABs age and androgen production decreases, an accelerated reduction of trabecular and cortical bone density occurs due to an increase in new osteoclast cells. If no therapies are provided, osteoporosis, as well as fractures of the newly weakened bones, can occur [3,7].

In AFABs with breast cancer, a variety of hormonal and nonhormonal treatments have the potential to cause hypogonadism as the treatments can produce an effect similar to menopause, regardless of age at the onset of treatment [4]. Hormonal treatments or endocrine therapies such as selective estrogen receptor modulators and nonhormonal therapies such as surgical procedures (i.e., a bilateral oophorectomy) can also result in hypogonadism and subsequent bone loss [4]. AMABs suffering from prostate cancer may choose to treat the disease through either surgical (i.e., bilateral orchiectomy) or medical castration, producing a similar bone loss effect [7,8].

Cancer is a leading cause of death worldwide, accounting for approximately 10 million deaths in 2020 and with millions of cases newly diagnosed each year [9]. While cancer and cancer treatment have the potential to cause BMD loss, it is unknown whether such changes impact the skeletal regions that undergo age-related changes and are used in forensic age estimation. Moreover, decedents with cancer are likely to be encountered in forensic cases. For example, in the New Mexico Decedent Image Database (NMDID)—a modern forensic sample from Albuquerque, NM, that was used in the present study—out of approximately 15,000 individuals, 4.2% (*n* = 633) had a record of cancer. As such, this study examines whether those with documented cancer exhibit advanced age-related changes to the pubic symphysis compared to those without documented cancer in a modern forensic sample. 

### 1.2. Pubic Symphysis Age-Estimation Methodology

One of the most used and well-known methods of aging individuals at death is the Suchey–Brooks method, which uses the degenerative nature of the pubic symphysis to produce an estimated age range of the individual [10]. Their study was completed on pubic bones (*n* = 1225) that were obtained from the Los Angeles County Coroner’s Office between 1977 and 1979, thereby representing the largest sample size of any previously published study of the pubic symphysis [10]. Sex, ancestry, and age-at-death were known and recorded for all individuals within the study; this information was provided by the coroner’s office and was confirmed by family members. Todd’s [11] original method of aging was applied to this new sample, and it was determined that some of the original ten phases and associated age ranges could be combined [10]. The authors subsequently created descriptions of six phases (I-VI) and created casts of the different phases for both AFABs and AMABs, which make this method easy to use for researchers at varying experience levels.

Hartnett [12] analyzed the Suchey–Brooks method using a more recent sample of individuals from modern forensic cases (*n* = 630) at the Maricopa County Forensic Science Center in Phoenix, Arizona. The sex, ancestry, and age-at-death of the individuals were known, and potential drug and alcohol abuse histories were obtained when possible. The author assigned a phase to each pubic symphysis using the Suchey–Brooks method and established that the method was moderately accurate in interobserver tests, with a higher accuracy reported for AFABs. The samples were then split into groups by sex and were further categorized based on a visual comparison of differing morphologies. Hartnett’s [12] method considered porosity, weight, and “feel,” which were the most important diagnostic markers for determining phases. However, these dry-bone markers cannot be assessed with CT data.

While CT machines designed for commercial use were not developed until 1971 [13], scanners have become more commonplace in research settings, specifically in forensic anthropology and age estimation [14]. For example, Telmon et al. [15] examined whether the Suchey–Brooks method was reliable when applied to CT scans. The authors used 21 pubic bones (7 dried and 14 with soft tissue) collected from autopsies at the Department of Forensic Medicine in Toulouse. The pubic symphyses with soft tissue still attached were CT-scanned in anatomical position and assigned a phase following the Suchey–Brooks method. After the scans were completed, the bones were macerated by placing them into warm water to remove the soft tissue. The authors then used the Suchey–Brooks method on the macerated bones and assigned a corresponding phase to each pubic symphysis. In the intraobserver study, phase assignments made by the more-experienced observer agreed 81% of the time, whereas the less-experienced second observer agreed 71% of the time. The authors found that there was overall good agreement between the use of dry-bone and CT scans, which suggested that CT scans were a reliable representation of the bone and showed the necessary features required for the method. 

Wink [16] furthered the study of pubic symphyses with CT scans by testing the reliability of the Suchey–Brooks method on a sample of CT scans from living individuals in the Northeastern United States. Wink [16] used clinical CT scans from the Boston University Medical Center in Massachusetts of both AFABs and AMABs (*n* = 44) between the ages of 19 and 89 years old. The author found that the Suchey–Brooks method correctly captured the true age of the individual 79.5% of the time in both of their trials. Individually, AMABs were assigned the correct phase 70.0% of the time, and AFABs were assigned the correct phase 87.5% of the time. In their intraobserver reliability study, the two tests were in perfect agreement 72.7% of the time. Wink [16] noted that in the cases where the correct age was not predicted, the errors occurred in both Phase III and Phase IV, which is consistent with previous findings [15]. The results of Wink’s [16] study suggest that CT scans are a valuable tool for forensic anthropology as the physical bone is not needed to estimate age using the pubic symphysis, which can be helpful for fleshed individuals or further research purposes. Moreover, CT scan-based studies of living individuals and/or deceased individuals allow for large sample sizes that encompass significant skeletal variation. 

While numerous studies have validated the Suchey–Brooks method [17,18,19], very few have included individuals with specific pathologies. Because one of the most popular areas on the skeleton to estimate age-at-death is the pubic symphysis and the most-preferred method is the Suchey–Brooks method [20], this method was used in the present study to test how the presence of a relatively common disease such as cancer can influence the method’s performance. Presently, no research exists on how cancers, and the associated BMD loss, can affect specific methods originally developed on “healthy,” non-pathological bone. If a decedent has a history of cancer and is found in a skeletonized state, the resulting BMD loss from cancer could potentially affect the accurate establishment of the biological profile, which may hinder identification.

## 2. Materials and Methods

This study examines whether the Suchey–Brooks pubic symphysis age estimation method can accurately estimate the age of individuals who have had cancer within their lifetime. The study sample was comprised of AFABs (*n* = 152) and AMABs (*n* = 155) from the NMDID, which is comprised of over 15,000 anonymized CT-scanned individuals who died in New Mexico between 2010 and 2017 [21,22]. The NMDID includes variably populated metadata for all individuals, such as medical histories, drug use, medications, age, ethnicity, cause of death, sex, and gender identities, among other demographic, health, and lifestyle data. Due to the anonymous nature of the decedents included in the NMDID, this project was given “exempt” status by Boston University’s Institutional Review Board (IRB exemption H-41202). 

### 2.1. Materials

Individuals with some form of documented cancer (e.g., breast, prostate, colorectal cancer, etc.) in their lifetime were selected from the NMDID to represent the study’s cancer cohort (AFAB = 90; AMAB = 92). Individuals with reported bone cancer such as osteosarcoma, Ewing sarcoma, and blood cancers like leukemia were excluded from the cancer group as the associated potential bony changes could skew results [23,24]. Individuals with cancers classified as specific tumors (i.e., brain tumors and pituitary tumors) were also excluded. While specific cancers were frequently documented in the accompanying metadata (4.2% of database), other information such as cancer treatments, active versus recovered cancer, and time since cancer diagnosis were not always available in the metadata. Thus, only the presence of cancer was used for the inclusion criteria. 

Additionally, those without documented cancer or other conditions that could impact BMD were selected as a comparative control cohort from the NMDID (AFAB = 62; AMAB = 63). As such, individuals with cancer, tuberculosis, hepatitis C, diabetes, chronic kidney disease, cirrhosis of the liver or liver disease, and death related to drug use or alcoholism were excluded from the control group as these diseases are known to decrease BMD [25,26,27,28,29,30,31,32,33]. While the NMDID metadata did not document cancer in the control cohort, it is possible that some individuals with cancer could have been included due to incomplete antemortem information for some NMDID individuals or due to an individual not being diagnosed with cancer prior to death.

To ensure that all age ranges were equally represented, the AFAB and AMAB groups were separated into age cohorts during data collection (Table 1). These groupings consist of ten-year age ranges with approximately 15 individuals in each age range. However, three age ranges have fewer than 15 individuals in both the AFAB and AMAB groups, which was due to a lack of very young and very old individuals in the database. The AFAB age range was 20–98 years, while the AMAB age range was 22–99 years. Table 2 shows the population affinity of the individuals included in this study. In most cases, the data in the NMDID are reported by the deceased’s next of kin to the Office of the Medical Investigator and therefore include more categories than the U.S. census classifications.

### 2.2. Methods

The CT scans were downloaded from the NMDID and were processed using the 64-bit OsiriX Imaging Software version 13.0.2 (Pixmeo, Geneva, Switzerland). OsiriX is an advanced open-source PACS workstation DICOM viewer (obtainable at www.osirix-viewer.com accessed on 10 July 2024). The software easily renders CT scans into a three-dimensional image that allows for further manipulation of the scans, including rotation, positioning, and surface- and volume-rendering capabilities. The three-dimensional images were created using the volume-rendering function with the “low-contrast” preset, as this was determined to be the best preset for viewing features of the pubic symphysis. However, in some cases, the “mid-contrast” preset was needed due to increased levels of porosity in some individuals. 

Scans isolating the torso were used and volume rendered—the arms (if visible), rib cage, spine, and sacrum were removed using the scissor tool. Next, the right os coxa was removed using the bone removal tool as only the left pubic symphysis was examined. Extraneous material in the scans, such as remaining soft tissue, contrast used for imaging, or medical equipment (e.g., catheters, intravenous lines, AED pads, etc.) on the body, was removed with the scissor tool.

The known age of each individual was recorded in a separate document for a later comparison of the estimated age range using the Suchey–Brooks method versus the chronological reported age. All scans were randomized for analysis, but the AFAB and AMAB scans were kept separate during the scoring process due to the finding that there are sex differences in pubic symphysis age-estimation methods and associated statistical applications [10,34,35,36]. The individuals were aged using the Suchey–Brooks method with the help of associated material such as the female and male casts and the original descriptions. The estimated phase was recorded in a separate document and compared to the individual’s chronological age after the completion of the study. An intraobserver study by the first author was conducted on 10% (*n* = 30) of the original study sample using a random selection of AFABs (*n* = 15) and AMABs (*n* = 15). This was completed several weeks after the original data collection. 

Descriptive statistics included in the original study were used for the statistical analysis [10]. Brooks and Suchey [10] reported a 95% age interval for each phase of their method including the mean age and standard deviation associated with each phase. An individual was considered correctly aged if their chronological age fell within the reported 95% range. A Mann–Whitney U test was conducted on AMAB cancer and control groups combined and AFAB cancer and control groups combined. A Kruskal–Wallis test with a Bonferroni post hoc test was conducted on the data to determine a relationship between the assigned phase and chronological age and to determine a relationship between the four cohorts (i.e., AFAB cancer, AFAB control, AMAB cancer, and AMAB control). A Cohen’s kappa test was conducted on the AFAB, AMAB, and combined AFAB and AMAB intraobserver study to test the intra-rater reliability of the Suchey–Brooks method. These statistical tests were completed in SPSS version 27.0.1.0 [37]. Further, the descriptive statistics of the groups are reported, including the percentage correct of each group (AMAB and AFAB) and a further breakdown into each category (cancer and control) and phase. If the wrong phase was assigned to an individual, the years from the closest end of the range are reported as well. 

## 3. Results

Once age estimations were generated using the pubic symphysis, the estimated age range was compared to the chronological age of the individuals at the time of death. A total of 25 AFABs and 7 AMABs could not be assessed for age estimation due to a fusion of the pubic symphysis, marked porosity, or a lack of CT scans despite their inclusion in the NMDID. This resulted in a final sample size of scans from 126 AFABs and 148 AMABs (*n* = 274). Individuals were considered aged correctly if their chronological age fell within the 95% prediction age range associated with each phase following Brooks and Suchey [10]. Individuals over the age of 87 years for AFABs and 86 years for AMABs that were estimated to be in Phase VI (latest phase) were considered correctly aged as this method does not include age estimations over these ages. 

For AFABs, combined cancer and control groups were aged correctly 78.6% of the time. Further breaking down the groups, AFABs with cancer were aged correctly 74.7% of the time, and AFABs without cancer were aged correctly 85.1% of the time (Table 3). For AMABs, combined cancer and control groups were aged correctly 50.0% of the time. AMABs with cancer were aged correctly 46.0% of the time, and AMABs without cancer were aged correctly 55.7% of the time (Table 3).

Table 4 presents a breakdown of AFAB cancer and control groups by phase, which shows the mean age and standard deviation for all phases. A standard deviation is not given for Phases I and II due to a lack of individuals assigned to those phases. The means were greater in the cancer group than the control group in Phases IV and V, but the standard deviations were larger in the control group. In both cancer and control groups, Phase III had the lowest percentage of individuals aged correctly; however, this phase only had a total of 13 individuals, which is considerably lower than the number of individuals in Phases IV, V, and VI. Similarly, Table 5 presents a breakdown of AMAB cancer and control groups by phase with the reported mean and standard deviations for each phase of this study group. A standard deviation was not reported for Phases I and II due to a lack of individuals assigned to these phases. The mean of the cancer group was larger than the control group in Phases III and VI, and the standard deviations were larger in Phases III, V, and VI. Phase II only includes a total of two individuals, with 50.0% (*n* = 1) aged correctly, and Phase III includes 15 individuals, with 26.6% (*n* = 4) aged correctly. These two groups had the fewest number of individuals in each of the phases. 

For all individuals whose age was underestimated, the years from the assigned range to the individual’s chronological age were calculated, and descriptive statistics were calculated (Table 6). For AFABs with cancer, the mean age above the incorrectly assigned age range was 13.47 years, and the AFAB control group had a mean age of 15.00 years. AMABs with cancer had a mean age of 15.51 years above the assigned age range, and the AMAB control group had a mean age of 16.15 years above the assigned range. An average for all groups combined was 15.26 years. All misclassifications except for four individuals—two AFABs with cancer, one AFAB without cancer, and one AMAB with cancer—were overestimations of the individual’s chronological age or the limits of the assigned age range were less than the individual’s chronological age.

### 3.1. Statistical Analyses

A Mann–Whitney U non-parametric test was conducted on AFABs and AMABs separately to determine any significance between cancer and control groups. For AFABs, even though the individuals with cancer were aged incorrectly more often than the control individuals, there was no statistical significance between the two groups (*p =* 0.170). Similarly, AMABs demonstrated a lack of statistical significance between the cancer and control groups (*p =* 0.422). A Kruskal–Wallis statistical analysis was run on all groups combined to determine significance between the means of the groups. A Bonferroni post hoc test was included in the analysis to determine exactly which groups were significant from each other. Statistical significance was determined between three groups: AFAB cancer and AMAB cancer (*p* = 0.001), AFAB control and AMAB cancer (*p* = 0.000), and AFAB control and AMAB control (*p* = 0.006). 

### 3.2. Intraobserver Error Tests

A total of 30 individuals were reanalyzed for the intraobserver error test (15 AFABs and 15 AMABs). In terms of intraobserver reliability, phase determinations between the first and second observations agreed 40.0% of the time for AFABs and 53.3% for AMABs. In some cases, different phases were assigned during the two analyses, but due to the large age ranges associated with the method, the individual’s chronological age was still accurately reported. This happened in 53.3% of cases for AFABs (*n* = 8) and 40.0% of cases for AMABs (*n* = 6). For the AFAB intraobserver error test, two individuals (13.3%) were aged one phase below the originally assigned phase, three individuals (20.0%) were aged one phase above the originally assigned phase, and two individuals (13.3%) were assigned a phase on the first test but were determined to be un-scorable the second time. For the AMAB intraobserver error test, three individuals (20.0%) were scored one phase below the originally assigned phase and three individuals (20.0%) were scored one phase above the originally assigned phase. 

A Cohen’s kappa test was run on the first and second intraobserver analyses to test the reliability and repeatability of the Suchey–Brooks method. For AFABs, the test reported a moderate intra-rater reliability (*k* = 0.587) following Landis and Koch [38], which was statistically significant (*p* = 0.013) and suggests that the Suchey–Brooks [10] method can be reliably repeated on a randomized group of individuals from this sample. For AMABs, the test similarly reported a moderate intra-rater reliability (*k* = 0.602) following Landis and Koch [38], which was statistically significant (*p* = 0.019) [38]. A Cohen’s kappa test on the combined AFAB and AMAB samples also resulted in a moderate intra-rater reliability (*k* = 0.600) following Landis and Koch [38], which was statistically significant (*p* < 0.001).

## 4. Discussion

This study aimed to test the reliability of the Suchey–Brooks method on a sample of CT scans from individuals who had experienced a form of cancer within their lifetime. The analysis of the three-dimensional reconstructions of pubic symphyses to estimate the age of individuals who had cancer yielded mixed results. The assigned age ranges captured the actual age (i.e., chronological age) of AMABs with cancer 46.0% of the time and the true age of AFABs with cancer 74.7% of the time. These results were lower than the control individuals, with AMABs correctly aged 55.7% of the time and AFABs aged correctly 85.1% of the time; however, these differences were not statistically significant. These results are most comparable to the results from Wink [16], who reported a higher accuracy rate for AFAB samples (87.5%) compared to the AMAB sample (70.0%). Hartnett [12] also reported that AFABs had a higher correlation rate in their interobserver tests than AMABs between three observers. 

All cases of incorrect age estimation were underestimations (average 15.26 years) of the chronological age, except for four cases that were over-aged (one cancer AMAB, one control AFAB, and two cancer AFABs). The AMAB with cancer was 12 years from the lowest age in the assigned age range (actual age: 22 years, assigned Phase: VI, associated age range: 34–86 years); the control AFAB was 5 years from the lowest age in the assigned age range (actual age: 20 years, assigned Phase: V, associated age range: 25–83 years); the first cancer AFAB was 3 years from the lowest age in the assigned age range (actual age: 39 years, assigned Phase: VI, associated age range: 42–87 years); and the other cancer AFAB was 1 year from the lowest age in the assigned age range (actual age: 41 years, assigned Phase: VI, associated age range: 42–87 years). Similar to the findings by Telmon et al. [15], the phases where most of the disagreements occurred were between Phases III and V. For both cancer AMABs and AFABs, most of the disagreements occurred in these phases, whereas the disagreements between chronological age and phase for control AMABs occurred in Phases IV and V and only in Phase IV for control AFABs. Telmon et al. [15] experienced both under- and over-aging of the individuals on CT scans—different from the findings of Wink’s [16] study, where all misclassifications of phase assignment were underestimations. These differences are unsurprising as Brooks and Suchey described a wide range of skeletal variation within these phases, which has been confirmed by other researchers [10,12,15,16,39]. 

The AMAB accuracy rate may be lower than the AFAB accuracy rate for a variety of reasons. One reason may be that the AFAB pubic symphysis has been described as aging more quickly or showing signs of degenerating faster than AMAB pubic symphyses [10,40,41]. This is also seen in the assigned age ranges for the Suchey–Brooks method, where AFAB age ranges are consistently higher than the age ranges assigned to AMABs of the same phase. The higher age ranges already seen in the AFAB phases seem to accommodate the degree of bone degeneration, if any, caused by cancer. The lower rate of accuracy in the AMAB control individuals in comparison to the AFAB control individuals is consistent with some researchers’ data on the accuracy rates of the Suchey–Brooks method [12,16,19,39], although some researchers have also demonstrated higher accuracy rates in AMABs [42,43].

**Figure 1:**
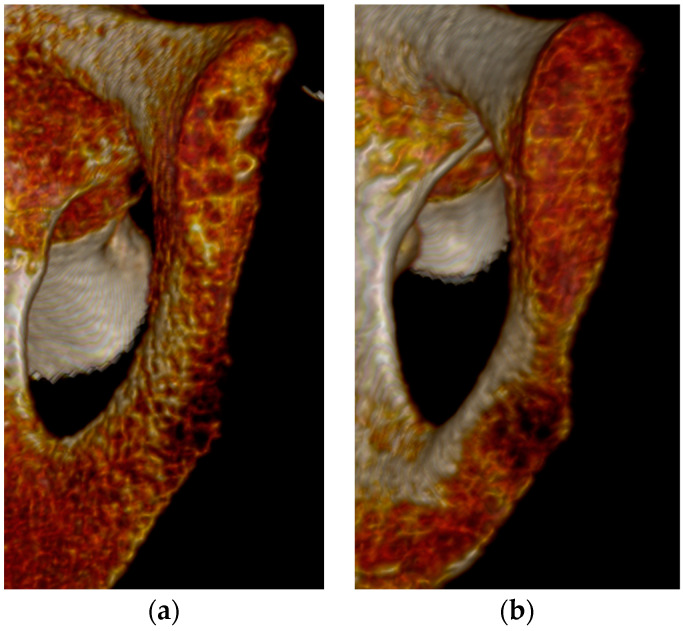
Suchey–Brooks Phase II left pubic symphysis. (**a**) AMAB aged 23 years, visualization of ridges and furrow on symphyseal face. (**b**) AFAB aged 36 years, visualization of ridges and furrows on symphyseal face.

**Figure 2:**
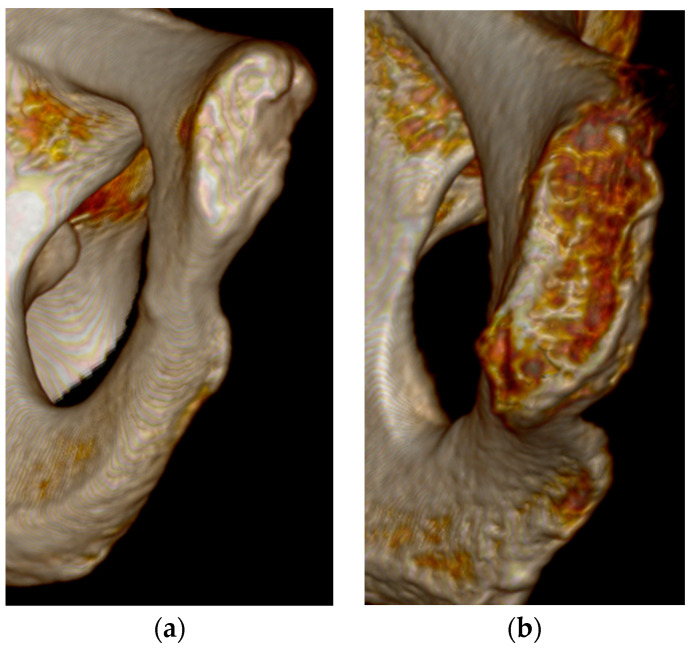
Suchey–Brooks Phase III left pubic symphysis. (**a**) AMAB aged 33 years, visualization of complete dorsal plateau. (**b**) AFAB aged 53 years, visualization of complete dorsal plateau.

**Figure 3:**
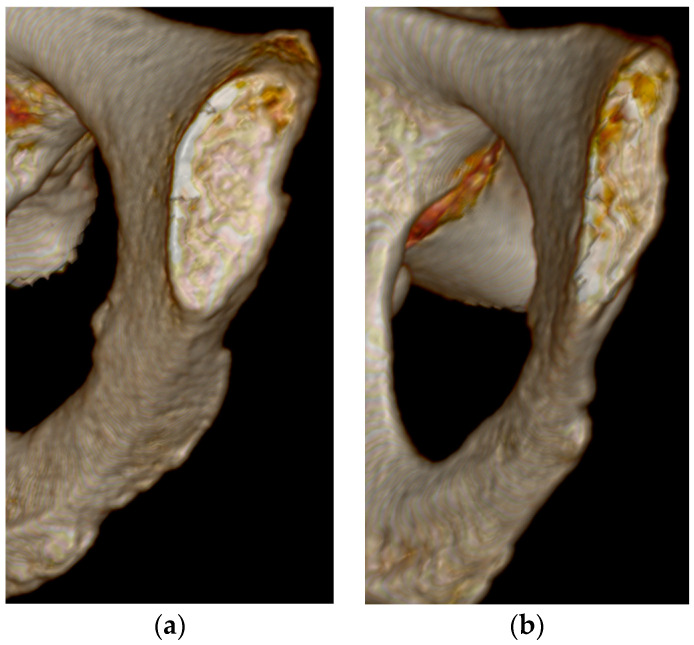
Suchey–Brooks Phase IV left pubic symphysis. (**a**) AMAB aged 48 years, visualization of complete oval outline. (**b**) AFAB aged 48 years, visualization of complete oval outline.

**Figure 4:**
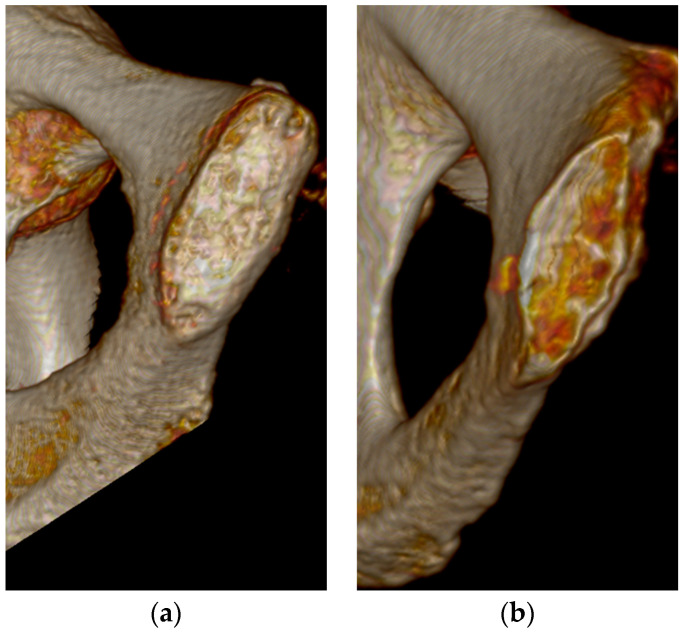
Suchey–Brooks Phase V left pubic symphysis. (**a**) AMAB aged 62 years, visualization of moderate lipping. (**b**) AFAB aged 54 years, visualization of slight depression of the face.

**Figure 5:**
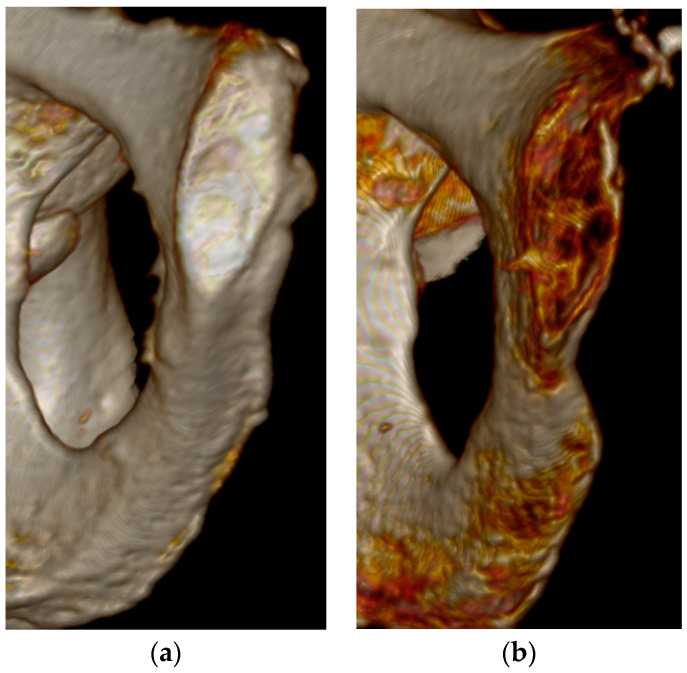
Suchey–Brooks Phase VI left pubic symphysis. (**a**) AMAB aged 58 years, visualization of depressed symphyseal face and ligamentous outgrowths. (**b**) AFAB aged 86 years, visualization of depressed symphyseal face and porosity.

The best overall features seen on the three-dimensional volume reconstructions of the pubic symphyses to estimate age were the symphyseal rim and lipping of the dorsal aspect. In some cases, a depression of the symphyseal face was hard to determine, especially during Phase V. There were no individuals assigned to Phase I in this study, but diagnostic features seen for Phase II were the ridges and furrows on the face and the incomplete rim (Figure 1). Individuals who were in Phase III were mostly categorized by a complete dorsal plateau with an almost completed ventral rampart and an absence of lipping (Figure 2). Phase IV was assigned to an individual using the features of a completed oval outline and slight lipping of the rim (Figure 3). Phase V was determined by a slight depression of the symphyseal face (although this was hard to see at times), slight porosity, and moderate lipping (Figure 4). In Phase VI, the depression was visible along with the lipping of the dorsal border, ventral ligamentous outgrowths, and an increase in porosity (Figure 5). Features that were not seen, or were not seen often, were ossific nodules in the earlier phases and large ventral ligamentous outgrowths that are typically seen on dry bone. It should be noted that all figures included are images of pubic symphyses from the study created by the authors.

Mann–Whitney U tests indicate no difference between AFAB and AMAB cancer and control cohorts; however, the presence of cancer appears to decrease the classification accuracy somewhat using the Suchey–Brooks method. These tests suggest that while there was a difference in the number of individuals that were correctly assigned the appropriate Suchey–Brooks phase in both AFABs and AMABs, cancer does not seem to influence BMD to the point where the Suchey–Brooks method cannot be reliably used on CT scans.

Kruskal–Wallis H tests were then run on all cohort combinations to determine if there was a significance between any of the groups. Of the six tests run, three groups were considered statistically significant after the Bonferroni post hoc test was applied to the data, which confirms the sex differences in correct phase scores (AFABs aged more accurately). These groups were AMAB cancer and AFAB cancer (*p* = 0.001), AMAB cancer and AFAB control (*p* = 0.000), and AMAB control and AFAB control (*p* = 0.006). Sexual differences in age-related changes of the pubic symphysis have been documented by numerous researchers [10,11,12,35,41,44]. However, because no differences were found between the AFAB cancer and control cohort or the AMAB cancer and control cohort, cancer does not seem to significantly affect the Suchey–Brooks method when used on CT-scanned individuals. Nevertheless, the analysis of CT scans precluded the assessment of bone “quality” and feel following Hartnett’s [12] amendments of the Suchey–Brooks method. 

The difference in correct classification rates between cancer and control groups—though not statistically significant—may be caused by a few different variables. Firstly, Brooks and Suchey [10] describe a wide range of variation within Phases III, IV, and V and the age ranges for each are very broad (e.g., Phase III age range is 21–53 years, Phase IV age range is 26–70 years, and Phase V age range is 25–83 years), which may account for some of the variations seen within the phases. In this study, AMABs had an overall lower correct phase assignment (50.0%) than AFABs (78.6%), which demonstrates that AMABs exhibit more variation in pubic symphysis age-related changes compared to AFABs [11,35,41,44]. While AFABs were aged more correctly than AMABs, this may also be attributed to the large age ranges associated with the upper phases for AFABs. These larger and older age ranges compared to the AMAB ranges may also be the reason that AFABs with cancer were aged more correctly than AMABs—the potential BMD loss and variation created within the pubic symphysis could be accounted for in the already larger age ranges provided. 

Second, BMD is known to decrease due to cancer and cancer treatments, as previously discussed [2,3,4,7,45,46,47]. The forensic anthropological methods created to estimate age from the pubic symphysis are based on the naturally degenerative nature of this area. If BMD decreases at an earlier age than previously established as the norm due to disease, this could potentially account for the lower accuracy rates of both AFABs and AMABs with cancer. The creation of this method excluding pathologies does not provide researchers with a complete understanding of how individuals age over time, especially with degenerative diseases such as cancer that affect more of the population today than in previous years [9]. The current anthropological methods of age assessment using bone degeneration force the assumption that only older individuals can experience bone loss, thus incorrectly aging individuals that experience BMD loss due to disease.

Lastly, another explanation for the lower accuracies in the AMAB cancer sample than both the AFAB samples and the AMAB control sample is that more individuals in the AMAB group were studied with the mid-contrast preset instead of the low-contrast preset. The low-contrast preset was used most often for examination, but individuals with more porosity were examined with the mid-contrast preset as this allowed for better visualization of the pubic symphysis. Even though it allowed for a better visualization of the pubic symphyses, almost every AMAB where this preset was used was assigned to a phase that underestimated their chronological age. 

Due to 85% of the combined AFAB and 90% of combined AMAB samples being comprised of white individuals, population differences were not examined in the present study. While race or ancestral identity cannot explain why there may be population differences in aging a sample from the US, racialized experiences and the embodiment of racism, including weathering, may impact overall skeletal health [48,49,50,51,52,53]. This, in turn, can impact the accuracy of forensic anthropological methods. Additionally, numerous studies have documented differing levels of accuracy when using the Brooks and Suchey method on non-US samples [17,18,19,42,43]. For example, Djurić et al. [42] reported accuracy rates for AMABs of 82.98% and 75.0% for AFABs from their Serbian sample groups, which, when compared to the original Suchey–Brooks study group, differed significantly. Kimmerle et al. [17] suggested that significant biological variation in the aging processes of different populations potentially resulting from environmental differences that affect the metabolic rate may affect the Serbian group and the American groups differently, which could account for biological differences that could alter the process of aging. 

The Suchey–Brooks method only ages AFABs up to 87 years and AMABs up to 86 years. Individuals in the sample were chosen from 20 years old to 99 years old as this represents a modern-day sample of living individuals. Many older individuals today live well past 87 years and many authors have called for better aging methods for older individuals [12,54,55]. Individuals that were over these age limits and categorized as Phase VI were considered correctly aged as there is no other advanced phase these individuals could be placed in. For AFABs, a total of 17 individuals over the age of 87 years were included in the study. Two were considered un-scorable due to high levels of porosity or complete fusion of the pubic symphyses and five (33.33%) were aged correctly (i.e., placed in Phase VI). For AMABs, a total of 15 individuals over the age of 86 years were included in the study. Three were un-scorable and only two (16.66%) were aged correctly. In reality, when working with older individuals in forensic cases, multiple methods would be used to develop an age estimation [56]. If individuals above the age limit were to be considered incorrectly aged, even if assigned to Suchey–Brooks Phase VI, Berg [54] or Hartnett’s [12] methods would be used as both methods add a Phase VII, which is useful in aging older individuals [12,54,57]. These methods were not used because the “feel,” relative weight, and quality of the bone are the most important variables for establishing age in Hartnett’s [12] method specifically. 

### 4.1. Limitations

While the Suchey–Brooks method is still the most widely used method of estimating age from the pubic symphysis [20], more research should be conducted on how and why this method varies for different demographics, including individuals with pathologies. Ancestry was not a factor in this research as much of the sample was white and socioeconomic status was not included, although this may be important in terms of the level and quality of treatment an individual received for their cancer. Moreover, all individuals included in this study lived in the United States and thereby broadly shared a North American environment. 

Overbury et al. [58] suggested that both right and left os coxae should be used when estimating age-at-death with the pubic symphysis, in case an individual shows extreme asymmetry. This was not done for this study as only the left os coxae were viewed to estimate age. Overbury et al. [58] described that asymmetry is very likely since the aging methods of the pelvis are based on highly degenerative features, which can deteriorate at different times due to genetic factors, environmental stressors, and certain biomechanical factors. The authors suggest that in cases of asymmetrical pubic symphyses, both sides should be aged, and the older of the two scores should then be used. With a disease such as cancer that can affect BMD and cause greater BMD loss than normal, tests of asymmetry should be completed to determine if both right and left os coxae degenerate similarly or if levels of asymmetry are higher in individuals with a disease that affects BMD. 

Another limitation of this study may be the small sample size in each of the four cancer/no cancer cohorts. AFABs with cancer started at 90 individuals and AFABs without cancer started at 62 individuals, though a combined total of 26 AFABs were unable to be scored. The AMAB cancer cohort started with 92 individuals and the AMAB group without cancer started 63 individuals, with only 7 total individuals unable to be scored between both groups. These groups are small compared to the 633 total individuals with cancer in their medical records in the NMDID, and this study could be expanded to include all individuals. 

### 4.2. Future Directions

It is recommended that future pubic symphysis aging techniques—and all techniques used to estimate age-at-death using the skeleton—should include pathological individuals. Pathologies, especially cancer, are relatively commonplace in society and are a normal aspect of any population, however defined. Excluding a wide array of pathological individuals, as is the status quo in methodological development, has the potential to create biased age-estimation methods that are not reflective of modern human skeletal variation. No group or population of individuals will be free of pathology, including those from forensic contexts. Moreover, disease status, including cancer diagnosis, will be unknown for the vast majority of forensic cases and therefore methods developed with a wide range of variation will be better suited for forensic casework. 

Some researchers have begun to create new quantitative methods for age estimation that are used with three-dimensional modeling to accurately predict the age of individuals [59]. While the Suchey–Brooks method produced moderate accuracy and reliability levels in the present study, newer methods that can be applied to CT scans to estimate age more precisely should be developed. One of the most difficult variables used to determine phase classification was porosity. In Phases V and VI, porosity is the main variable used to determine the phase on dry bone. On the CT scans, when a pubic symphysis was extremely porous, it was harder to determine the phase because the nuances of the symphyseal face were not obvious. To counteract this, the mid-contrast setting was used, but when this setting was applied, individuals tended to be incorrectly underaged. This may be because the mid-contrast levels showed the bone to be denser than it was. Another variable of the Suchey–Brooks method that could not be used was the relative weight and “feel” of the bone. Brooks and Suchey [10] and Hartnett [12] determined that these elements, along with porosity, are the most important variables when determining age-at-death for the later phases. A quantitative method like the one developed by Slice and Algee-Hewitt [59] or a component-based method like the one developed by Dudzik and Langley [60], which could be formatted to be used on CT scans, may be more effective at aging CT-scanned individuals. The use of CT scans in forensic medicine and forensic anthropology will only increase as the technology becomes more readily available to researchers. CT scans are non-invasive and non-destructive and can be applied to previously collected clinical data on living individuals, thereby increasing variation and sample sizes.

## 5. Conclusions

This study was the first to test the reliability of the Suchey–Brooks method on a sample of CT scans from individuals who had documented cancer within their lifetime and those without; this yielded mixed results. While those with cancer exhibited lower age classification accuracies, statistically, the Suchey–Brooks method was not significantly affected by BMD loss associated with cancer and/or cancer treatments, although this may be related to small sample sizes in each of the four groups. 

While not statistically significant, the presence of cancer does seem to impact the ability to accurately estimate age at death using the Suchey–Brooks method. More methods should be created for individuals that have pathologies, or methods should be created including these individuals instead of excluding them. Many different diseases can contribute to BMD loss, and these methods should take that into consideration. It is clear that individuals with a disease that can degenerate BMD prematurely, such as cancer, have not been included in the formation of the method, and the degree to which cancer can degenerate BMD is outside of the normal ranges of decreased BMD with age.

Furthermore, as technology and science progresses and reliable equipment becomes more accessible, methods that are specific to or modified for CT scans should be developed. CT scans have proven to be a useful tool in the study of individuals after death, whether the remains are skeletonized or fleshed. Further developing new methods to be used with this technology increases the chances that more individuals will be correctly identified in a forensic setting through accurate biological profile creation, also resulting in more accurate methods as sample sizes can be exponentially larger.

## Figures and Tables

**Table 1:** Distribution of the study sample by age, sex, and cancer presence/absence.

Group	20–29	30–39	40–49	50–59	60–69	70–79	80–89	90–99	Total
AFAB Cancer	1	10	15	15	15	15	14	5	90
AFAB Control	4	5	10	10	10	10	8	5	62
AMAB Cancer	4	8	15	15	15	15	15	5	92
AMAB Control	4	5	10	10	10	10	10	4	63
Total	13	28	50	50	50	50	47	19	307

**Table 2:** AFAB and AMAB distribution of study sample by population affinity.

Population	AFAB	AMAB
White (not Hispanic or Latino)	99 (65%)	104 (67%)
White (Hispanic or Latino)	32 (21%)	35 (23%)
Native American	10 (6%)	4 (2%)
Hispanic	6 (4%)	8 (5%)
Black or African American	3 (2%)	3 (2%)
Japanese	1 (1%)	0
Vietnamese	0	1 (1%)
“Other Asian”	1 (1%)	0

**Table 3:** AFAB and AMAB cancer, control, and combined correct classifications.

Group	Correct	Incorrect	Total
AFAB Cancer	59 (74.7%)	20 (25.3%)	79
AFAB Control	40 (85.1%)	7 (14.9%)	47
AFAB Combined	99 (78.6%)	27 (21.4%)	126
AMAB Cancer	40 (46.0%)	47 (54.0%)	87
AMAB Control	34 (55.7%)	27 (44.3%)	61
AMAB Combined	74 (50.0%)	74 (50.0%)	148

**Table 4:** Descriptive statistics for the AFAB cancer, control, and combined groups.

	Cancer			No Cancer			Total		
Phase	Mean	*n*	SD	Mean	*n*	SD	Mean	*n*	SD
I	-	-	-	20.0	1	-	20.0	1	-
II	-	-	-	36.0	1	-	36.0	1	-
III	61.1	11	19.7	70.0	2	24.0	62.5	13	19.6
IV	55.3	29	15.4	54.3	24	17.9	54.9	63	16.3
V	70.0	19	18.1	56.5	12	18.3	62.1	31	18.4
VI	68.1	10	18.1	73.1	7	20.8	70.2	17	18.8

**Table 5:** Descriptive statistics for the AMAB cancer, control, and combined groups.

	Cancer			No Cancer			Total		
Phase	Mean	*n*	SD	Mean	*n*	SD	Mean	*n*	SD
I	-	-	-	-	-	-	-	-	-
II	36.0	1	-	23.0	1	-	29.1	2	9.2
III	58.3	11	15.6	55.0	4	13.7	58.1	15	14.9
IV	60.4	52	16.8	57.5	35	17.1	59.2	87	16.9
V	65.2	25	20.5	69.5	16	17.4	66.9	41	19.2
VI	68.8	8	22.7	81.2	5	20.4	73.5	13	21.9

**Table 6:** Descriptive statistics for all incorrectly aged individuals.

Group	*n*	Mean	Median	Range	Outliers
AFAB Cancer	19	13.47	13	3–20	41
AFAB Control	6	15.0	13	4–20	34
AMAB Cancer	46	15.51	15	1–39	48
AMAB Control	27	16.15	16	1–26	38

## Data Availability

The original data presented in the study are openly available in the NMDID (https://nmdid.unm.edu/ accessed on 10 July 2024).
